# *rac*-*N*-[Hy­droxy(4-pyrid­yl)meth­yl]picolinamide: a hemiamidal

**DOI:** 10.1107/S1600536810021756

**Published:** 2010-06-16

**Authors:** Muhammad Altaf, Helen Stoeckli-Evans

**Affiliations:** a Institute of Physics, University of Neuchâtel, rue Emile-Argand 11, CH-2009 Neuchâtel, Switzerland

## Abstract

The title compound, C_12_H_11_N_3_O_2_, a hemiamidal, was synthesized by solvent-free aldol condensation at room temperature by grinding picolinamide with isonicotinaldehyde in a 1:1 molar ratio. In the mol­ecule, the two pyridine rings are inclined to one another by 58.75 (6)°. They are linked, at positions 2 and 4, by the hemiamidal bridge (–CO—NH—CHOH–). The NH-group H atom forms an intra­molecular hydrogen bond with the N atom of the picolinamide pyridine ring. In the crystal, symmetry-related mol­ecules are linked *via* N—H⋯O hydrogen bonds, involving the NH group H atom of the hemiamidal bridge and the hy­droxy O atom, forming inversion-related dimers, with graph-set *R*_2_^2^(8). Adjacent mol­ecules are also linked *via* O—H⋯N hydrogen bonds, involving the hy­droxy substituent and the 4-pyridine N atom. Together these inter­actions lead to the formation of double-stranded ribbon-like hydrogen-bonded polymers propagating in [010]. The latter are further connected *via* C—H⋯O hydrogen bonds involving the carbonyl O atom, so forming a two-dimensional network in (011).

## Related literature

For background to green synthesis, see: Raston & Scott (2000 ▶). For solid-state reactions and solvent-free syntheses, see: Kaupp (2003 ▶, 2005 ▶). For the structure of a similar non-cyclic hemiamidal, see: Kawahara *et al.* (1992 ▶). For details of the Cambridge Structural Database, see: Allen (2002 ▶). For standard bond lengths, see: Allen *et al.* (1987 ▶). For details of hydrogen-bonding graph-set analysis, see: Bernstein *et al.* (1995 ▶). For the illustration and analysis of hydrogen bonding, see: Macrae *et al.* (2006 ▶).
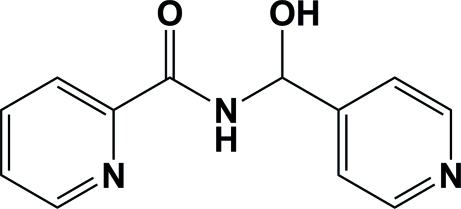


## Experimental

### 

#### Crystal data

C_12_H_11_N_3_O_2_
*M* *_r_* = 229.24
Monoclinic, 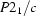

*a* = 12.1450 (13) Å
*b* = 5.6044 (4) Å
*c* = 16.3940 (19) Å
β = 111.354 (9)°
*V* = 1039.26 (18) Å^3^
*Z* = 4
Mo *K*α radiation
μ = 0.10 mm^−1^
*T* = 173 K
0.50 × 0.41 × 0.10 mm

#### Data collection

Stoe IPDS-2 diffractometer
14414 measured reflections
2823 independent reflections
2196 reflections with *I* > 2σ(*I*)
*R*_int_ = 0.079

#### Refinement

*R*[*F* ^2^ > 2σ(*F* ^2^)] = 0.042
*wR*(*F* ^2^) = 0.111
*S* = 1.04
2823 reflections
163 parameters
H atoms treated by a mixture of independent and constrained refinement
Δρ_max_ = 0.37 e Å^−3^
Δρ_min_ = −0.22 e Å^−3^

### 

Data collection: *X-AREA* (Stoe & Cie, 2004 ▶); cell refinement: *X-AREA*; data reduction: *X-RED32* (Stoe & Cie, 2004 ▶); program(s) used to solve structure: *SHELXS97* (Sheldrick, 2008 ▶); program(s) used to refine structure: *SHELXL97* (Sheldrick, 2008 ▶); molecular graphics: *PLATON* (Spek, 2009 ▶) and *Mercury* (Macrae *et al.*, 2006 ▶); software used to prepare material for publication: *SHELXL97* and *PLATON*.

## Supplementary Material

Crystal structure: contains datablocks I, global. DOI: 10.1107/S1600536810021756/lh5064sup1.cif

Structure factors: contains datablocks I. DOI: 10.1107/S1600536810021756/lh5064Isup2.hkl

Additional supplementary materials:  crystallographic information; 3D view; checkCIF report

## Figures and Tables

**Table 1:** Hydrogen-bond geometry (Å, °)

*D*—H⋯*A*	*D*—H	H⋯*A*	*D*⋯*A*	*D*—H⋯*A*
N2—H2*N*⋯N1	0.875 (17)	2.468 (18)	2.7769 (16)	101.4 (13)
N2—H2*N*⋯O2^i^	0.875 (17)	2.091 (17)	2.9304 (15)	160.7 (16)
O2—H2*O*⋯N1^ii^	0.905 (19)	1.92 (2)	2.8056 (16)	167.6 (17)
C7—H7⋯O1^iii^	1.00	2.43	3.3694 (15)	157
C12—H12⋯O1^iv^	0.95	2.43	3.3517 (17)	163

Symmetry codes: (i) 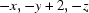; (ii) 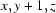; (iii) 
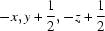
; (iv) 
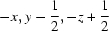
.

**Fig. 1.**
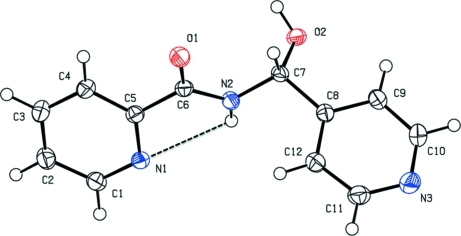
A view of the molecular structure of the title molecule, with dispacement ellipsoids drawn at the 50% probability level [The intramolecular N—H···N hydrogen bond is shown as a dashed line].

**Fig. 2.**
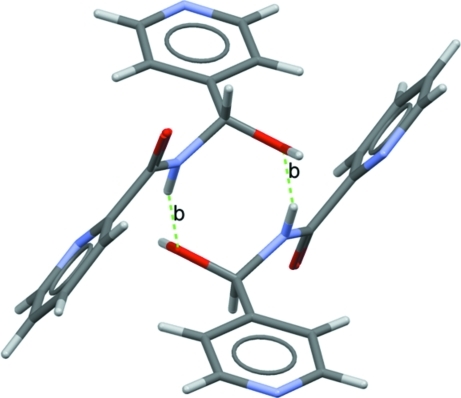
A view of the hydrogen bonded inversion dimers in the crystal of the title compound; graph-set R22(8)>b>b (Mercury: Macrae et al., 2006).

**Fig. 3.**
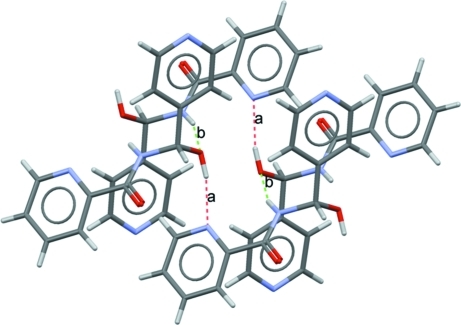
A view of the hydrogen bonded R44(14)a>b>a>b graph-set, involving adjacent molecules and those related by an inversion center, in the crystal structure of the title compound (Mercury: Macrae et al., 2006).

**Fig. 4.**
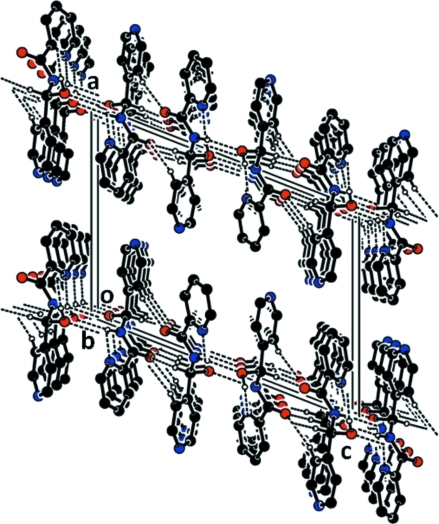
A view along the b-axis of the crystal packing of the title compound, with the N—H···O, O—H···N and C—H···O hydrogen bonds shown as dashed lines (see Table 1 for details).

## supplementary crystallographic information

### Comment

Green chemistry is a well established field of research, enhanced by its numerous applications in high technology industries and because of the need for environmentally friendly syntheses. The perfect *green reaction* has been described as one which: proceeds at room temperature, requires no organic solvent, is highly selective and exhibits high atom efficiency, yet produces no waste products (Raston & Scott, 2000). In recent decades, numerous reactions using mechanical activation have been reported to give 100% yield (Kaupp, 2005). This procedure has a number of advantages, such as rapid and qualitatively solvent free synthesis and no necessary work up procedure (Kaupp, 2003). On the other hand, there are some disadvantages, such as the use of harmful sodium hydroxide, or other sodium salts, as intermediates in the reaction work up. The title compound was synthesized by grinding picolinamide with isonicotinaldehyde in a molar ratio of 1:1 giving a brown gelatinous material. On drying in air a brown microcrystalline powder was obtained, which proved to be the title compound. The compound is a result of the combination of green chemistry and mechanical activation.

The molecule structure of the title molecule is illustrated in Fig. 1. The bond distances are normal (Allen *et al.*, 1987) and the bond angles in the hemiamidal bridge (–CO—NH-CHOH–) are similar to those observed in (1-hydroxybut-2-yl)ammonium *N*-benzoyl-α-hydroxyglycine (Kawahara *et al.*, 1992). Interestingly, here the authors showed that Peptidylglycine-hydroxylating monooxygenase (PHM), converted *N*-benzoylglycine stereoselectively to *S*-*N*-benzoyl-hydroxyglycine. The latter is the only non-cyclic example of such a (–CO—NH-CHOH–) unit that was found during a search of the Cambridge Crystal Structure Database (CSD, V5.31, last update May 2010; Allen *et al.*, 2002).

In the title molecule the two pyridine rings are inclined to one another by 58.75 (6) °, and the NH H-atom (H2N) forms an intramolecular hydrogen bond with the picolinamide N-atom (N1) (Fig. 1 and Table 1).

In the crystal molecules are linked *via* N—H···O and O—H···O hydrogen bonds (Table 1). The N—H···O hydrogen bonds, involving the hemiamidal NH group and the hydroxyl O-atom, lead to the formation of inversion dimers, graph-set *R*^2^_2_(8) (Fig. 2) (Bernstein *et al.*, 1995). Adjacent molecules are linked *via* O—H···N hydrogen bonds involving the hydroxyl H-atom (H2O) and the adjacent pyridine N-atom, N2 (Table 1). Together with molecules related by an inversion center these interactions lead to a graph-set of *R*^4^_4_(14) (Fig. 3). Finally these hydrogen bonding interactions lead to the formation of double-stranded ribbon-like polymers propagating in [010]. They are further linked *via* C—H···O interactions, involving the C═O O-atom, leading to the formation of two dimensional networks stacking along [100] (Fig. 4 and Table 1).

### Experimental

The synthesis of the title compound was carried out by mixing by hand in a mortar a 1:1 molar ratio of picolinamide with isonicotinaldehyde. A viscous brown mixture was obtained which on drying in air gave a brown micro-crystalline powder (Yield 98.78%). Elemental analysis (%) for C_12_H_11_N_3_O_2_: C, 62.88; H, 4.80; N, 18.34. Found: C, 62.75; H, 4.91; N, 18.54. IR (cm^-1^): 3471.51, 3288.31, 3025.77, 1676.26, 1616.03, 1590.90, 1569.42, 1412.63, 1156.79, 922.55, 747.07, 581.52. Crystals suitable for X-ray analysis were obtained during a failed attempt of complex formation with MnCl_2_.6H_2_O in a methanol/water solution. The colourless reaction solution obtained was filtered and the filtrate left undisturbed at RT for slow evaporation. After five days colourless plate-like crystals, suitable for X-ray diffraction analysis, were obtained.

### Refinement

The NH and OH H-atoms were located in a difference electron-density map and were freely refined: O—H = 0.905 (19) Å, N—H = 0.875 (17) Å. The C-bound H-atoms were included in calculated positions and treated as riding atoms: C—H = 0.95 Å for H-aromatic, and 1.00 Å for H-methine, with *U*_iso_(H) = 1.2*U*_eq_(C).

### Crystal data

**Table:**

C_12_H_11_N_3_O_2_	*F*(000) = 480
*M**_r_* = 229.24	*D*_x_ = 1.465 Mg m^−^^3^
Monoclinic, *P*2_1_/*c*	Mo *K*α radiation, λ = 0.71073 Å
Hall symbol: -P 2ybc	Cell parameters from 10944 reflections
*a* = 12.1450 (13) Å	θ = 1.8–29.6°
*b* = 5.6044 (4) Å	µ = 0.10 mm^−^^1^
*c* = 16.3940 (19) Å	*T* = 173 K
β = 111.354 (9)°	Plate, colourless
*V* = 1039.26 (18) Å^3^	0.50 × 0.41 × 0.10 mm
*Z* = 4	

### Data collection

**Table:**

Stoe IPDS-2 diffractometer	2196 reflections with *I* > 2σ(*I*)
Radiation source: fine-focus sealed tube	*R*_int_ = 0.079
graphite	θ_max_ = 29.3°, θ_min_ = 1.8°
phi + ω rotation scans	*h* = −16→16
14414 measured reflections	*k* = −7→7
2823 independent reflections	*l* = −22→22

### Refinement

**Table:**

Refinement on *F*^2^	Secondary atom site location: difference Fourier map
Least-squares matrix: full	Hydrogen site location: inferred from neighbouring sites
*R*[*F*^2^ > 2σ(*F*^2^)] = 0.042	H atoms treated by a mixture of independent and constrained refinement
*wR*(*F*^2^) = 0.111	*w* = 1/[σ^2^(*F*_o_^2^) + (0.0672*P*)^2^] where *P* = (*F*_o_^2^ + 2*F*_c_^2^)/3
*S* = 1.04	(Δ/σ)_max_ < 0.001
2823 reflections	Δρ_max_ = 0.37 e Å^−^^3^
163 parameters	Δρ_min_ = −0.22 e Å^−^^3^
0 restraints	Extinction correction: *SHELXL97* (Sheldrick, 2008), Fc^*^=kFc[1+0.001xFc^2^λ^3^/sin(2θ)]^-1/4^
Primary atom site location: structure-invariant direct methods	Extinction coefficient: 0.020 (4)

### Special details

**Table:**

Geometry. Bond distances, angles *etc*. have been calculated using the rounded fractional coordinates. All su's are estimated from the variances of the (full) variance-covariance matrix. The cell e.s.d.'s are taken into account in the estimation of distances, angles and torsion angles
Refinement. Refinement of *F*^2^ against ALL reflections. The weighted *R*-factor *wR* and goodness of fit *S* are based on *F*^2^, conventional *R*-factors *R* are based on *F*, with *F* set to zero for negative *F*^2^. The threshold expression of *F*^2^ > σ(*F*^2^) is used only for calculating *R*-factors(gt) *etc*. and is not relevant to the choice of reflections for refinement. *R*-factors based on *F*^2^ are statistically about twice as large as those based on *F*, and *R*- factors based on ALL data will be even larger.

### Fractional atomic coordinates and isotropic or equivalent isotropic displacement parameters (Å^2^)

**Table:**

	*x*	*y*	*z*	*U*_iso_*/*U*_eq_	
O1	−0.08117 (8)	1.01540 (16)	0.21882 (6)	0.0269 (3)	
O2	0.03250 (8)	1.27719 (16)	0.06003 (6)	0.0233 (2)	
N1	−0.15978 (9)	0.51231 (18)	0.08046 (7)	0.0216 (3)	
N2	−0.01442 (9)	0.91083 (19)	0.11117 (7)	0.0206 (3)	
N3	0.42324 (10)	0.8732 (2)	0.16564 (8)	0.0328 (3)	
C1	−0.24525 (11)	0.3491 (2)	0.05116 (9)	0.0253 (3)	
C2	−0.35054 (11)	0.3619 (3)	0.06494 (9)	0.0293 (4)	
C3	−0.36862 (11)	0.5519 (3)	0.11228 (10)	0.0299 (4)	
C4	−0.28077 (11)	0.7220 (2)	0.14387 (9)	0.0254 (3)	
C5	−0.17819 (10)	0.6966 (2)	0.12589 (8)	0.0206 (3)	
C6	−0.08534 (10)	0.8875 (2)	0.15735 (8)	0.0204 (3)	
C7	0.06900 (10)	1.1054 (2)	0.12781 (8)	0.0195 (3)	
C8	0.19102 (10)	1.0180 (2)	0.13730 (7)	0.0202 (3)	
C9	0.26572 (11)	1.1592 (2)	0.11155 (9)	0.0256 (3)	
C10	0.37952 (12)	1.0795 (3)	0.12702 (10)	0.0318 (4)	
C11	0.34982 (12)	0.7394 (2)	0.19001 (9)	0.0298 (4)	
C12	0.23415 (11)	0.8019 (2)	0.17731 (9)	0.0258 (3)	
H1	−0.23290	0.21720	0.01910	0.0300*	
H2	−0.40940	0.24250	0.04240	0.0350*	
H2N	−0.0255 (14)	0.824 (3)	0.0645 (11)	0.026 (4)*	
H2O	−0.0220 (16)	1.371 (3)	0.0699 (12)	0.038 (5)*	
H3	−0.44020	0.56550	0.12300	0.0360*	
H4	−0.29040	0.85360	0.17720	0.0310*	
H7	0.07430	1.18470	0.18380	0.0230*	
H9	0.23950	1.30840	0.08370	0.0310*	
H10	0.42980	1.17870	0.10890	0.0380*	
H11	0.37860	0.59140	0.21790	0.0360*	
H12	0.18560	0.69880	0.19570	0.0310*	

### Atomic displacement parameters (Å^2^)

**Table:**

	*U*^11^	*U*^22^	*U*^33^	*U*^12^	*U*^13^	*U*^23^
O1	0.0345 (5)	0.0250 (5)	0.0274 (5)	−0.0026 (4)	0.0186 (4)	−0.0048 (4)
O2	0.0272 (4)	0.0201 (4)	0.0265 (4)	0.0048 (3)	0.0145 (4)	0.0036 (3)
N1	0.0231 (5)	0.0199 (5)	0.0238 (5)	0.0013 (4)	0.0108 (4)	0.0005 (4)
N2	0.0236 (5)	0.0197 (5)	0.0209 (5)	−0.0023 (4)	0.0110 (4)	−0.0025 (4)
N3	0.0246 (5)	0.0305 (6)	0.0421 (7)	−0.0003 (4)	0.0107 (5)	−0.0041 (5)
C1	0.0276 (6)	0.0210 (6)	0.0275 (6)	−0.0008 (5)	0.0104 (5)	−0.0002 (5)
C2	0.0250 (6)	0.0279 (7)	0.0342 (7)	−0.0042 (5)	0.0100 (5)	0.0012 (5)
C3	0.0235 (6)	0.0322 (7)	0.0377 (7)	0.0003 (5)	0.0156 (5)	0.0025 (6)
C4	0.0255 (6)	0.0253 (6)	0.0290 (6)	0.0022 (5)	0.0141 (5)	0.0002 (5)
C5	0.0236 (5)	0.0197 (5)	0.0205 (5)	0.0026 (4)	0.0104 (4)	0.0036 (4)
C6	0.0231 (5)	0.0187 (5)	0.0213 (5)	0.0028 (4)	0.0103 (4)	0.0019 (4)
C7	0.0236 (5)	0.0167 (5)	0.0208 (5)	−0.0007 (4)	0.0111 (4)	−0.0009 (4)
C8	0.0220 (5)	0.0203 (6)	0.0191 (5)	−0.0025 (4)	0.0086 (4)	−0.0029 (4)
C9	0.0270 (6)	0.0222 (6)	0.0293 (6)	−0.0034 (5)	0.0124 (5)	0.0002 (5)
C10	0.0257 (6)	0.0312 (7)	0.0418 (8)	−0.0051 (5)	0.0162 (6)	−0.0008 (6)
C11	0.0270 (6)	0.0249 (6)	0.0339 (7)	0.0029 (5)	0.0069 (5)	−0.0007 (5)
C12	0.0264 (6)	0.0231 (6)	0.0279 (6)	−0.0005 (5)	0.0099 (5)	0.0017 (5)

### Geometric parameters (Å, °)

**Table:**

O1—C6	1.2226 (15)	C7—C8	1.5141 (18)
O2—C7	1.4140 (15)	C8—C9	1.3807 (18)
O2—H2O	0.905 (19)	C8—C12	1.3861 (16)
N1—C1	1.3348 (17)	C9—C10	1.385 (2)
N1—C5	1.3389 (16)	C11—C12	1.388 (2)
N2—C7	1.4452 (16)	C1—H1	0.9500
N2—C6	1.3443 (17)	C2—H2	0.9500
N3—C10	1.332 (2)	C3—H3	0.9500
N3—C11	1.3328 (19)	C4—H4	0.9500
N2—H2N	0.875 (17)	C7—H7	1.0000
C1—C2	1.379 (2)	C9—H9	0.9500
C2—C3	1.382 (2)	C10—H10	0.9500
C3—C4	1.383 (2)	C11—H11	0.9500
C4—C5	1.3881 (19)	C12—H12	0.9500
C5—C6	1.5029 (17)		
			
C7—O2—H2O	107.2 (11)	N3—C10—C9	124.42 (14)
C1—N1—C5	117.60 (12)	N3—C11—C12	124.34 (11)
C6—N2—C7	121.06 (10)	C8—C12—C11	118.62 (12)
C10—N3—C11	115.87 (13)	N1—C1—H1	118.00
C7—N2—H2N	117.3 (12)	C2—C1—H1	118.00
C6—N2—H2N	120.8 (12)	C1—C2—H2	121.00
N1—C1—C2	123.46 (12)	C3—C2—H2	121.00
C1—C2—C3	118.60 (14)	C2—C3—H3	121.00
C2—C3—C4	118.87 (13)	C4—C3—H3	121.00
C3—C4—C5	118.65 (12)	C3—C4—H4	121.00
N1—C5—C4	122.81 (11)	C5—C4—H4	121.00
C4—C5—C6	118.26 (11)	O2—C7—H7	108.00
N1—C5—C6	118.92 (11)	N2—C7—H7	108.00
N2—C6—C5	115.50 (11)	C8—C7—H7	108.00
O1—C6—C5	120.06 (12)	C8—C9—H9	121.00
O1—C6—N2	124.37 (12)	C10—C9—H9	121.00
O2—C7—N2	111.58 (10)	N3—C10—H10	118.00
O2—C7—C8	108.43 (10)	C9—C10—H10	118.00
N2—C7—C8	111.59 (10)	N3—C11—H11	118.00
C9—C8—C12	117.93 (12)	C12—C11—H11	118.00
C7—C8—C9	120.71 (10)	C8—C12—H12	121.00
C7—C8—C12	121.23 (11)	C11—C12—H12	121.00
C8—C9—C10	118.82 (12)		
			
C5—N1—C1—C2	0.34 (19)	N1—C5—C6—O1	−158.19 (12)
C1—N1—C5—C4	0.59 (18)	N1—C5—C6—N2	24.74 (16)
C1—N1—C5—C6	−178.08 (11)	C4—C5—C6—O1	23.08 (17)
C7—N2—C6—O1	−4.40 (19)	C4—C5—C6—N2	−153.99 (12)
C7—N2—C6—C5	172.53 (10)	O2—C7—C8—C9	25.04 (15)
C6—N2—C7—O2	−107.33 (13)	O2—C7—C8—C12	−159.37 (11)
C6—N2—C7—C8	131.21 (12)	N2—C7—C8—C9	148.32 (11)
C11—N3—C10—C9	0.1 (2)	N2—C7—C8—C12	−36.09 (15)
C10—N3—C11—C12	0.2 (2)	C7—C8—C9—C10	175.51 (12)
N1—C1—C2—C3	−0.7 (2)	C12—C8—C9—C10	−0.22 (19)
C1—C2—C3—C4	0.1 (2)	C7—C8—C12—C11	−175.24 (11)
C2—C3—C4—C5	0.7 (2)	C9—C8—C12—C11	0.47 (18)
C3—C4—C5—N1	−1.1 (2)	C8—C9—C10—N3	−0.1 (2)
C3—C4—C5—C6	177.56 (12)	N3—C11—C12—C8	−0.5 (2)

### Hydrogen-bond geometry (Å, °)

**Table:**

*D*—H···*A*	*D*—H	H···*A*	*D*···*A*	*D*—H···*A*
N2—H2N···N1	0.875 (17)	2.468 (18)	2.7769 (16)	101.4 (13)
N2—H2N···O2^i^	0.875 (17)	2.091 (17)	2.9304 (15)	160.7 (16)
O2—H2O···N1^ii^	0.905 (19)	1.92 (2)	2.8056 (16)	167.6 (17)
C7—H7···O1^iii^	1.00	2.43	3.3694 (15)	157
C12—H12···O1^iv^	0.95	2.43	3.3517 (17)	163

Symmetry codes: (i) −*x*, −*y*+2, −*z*; (ii) *x*, *y*+1, *z*; (iii) −*x*, *y*+1/2, −*z*+1/2; (iv) −*x*, *y*−1/2, −*z*+1/2.
